# Alternative Polyadenylation Allows Differential Negative Feedback of Human miRNA miR-579 on Its Host Gene ZFR

**DOI:** 10.1371/journal.pone.0121507

**Published:** 2015-03-23

**Authors:** Ludwig Christian Hinske, Pedro A. F. Galante, Elisabeth Limbeck, Patrick Möhnle, Raphael B. Parmigiani, Lucila Ohno-Machado, Anamaria A. Camargo, Simone Kreth

**Affiliations:** 1 Research Group Molecular Medicine, Department of Anaesthesiology, Clinic of the University of Munich, Munich, Germany; 2 Molecular Oncology Center, Sírio Libanês Hospital, São Paulo, Brazil; 3 Division of Biomedical Informatics, University of California San Diego, La Jolla, California, United States of America; University of Hong Kong, HONG KONG

## Abstract

About half of the known miRNA genes are located within protein-coding host genes, and are thus subject to co-transcription. Accumulating data indicate that this coupling may be an intrinsic mechanism to directly regulate the host gene’s expression, constituting a negative feedback loop. Inevitably, the cell requires a yet largely unknown repertoire of methods to regulate this control mechanism. We propose APA as one possible mechanism by which negative feedback of intronic miRNA on their host genes might be regulated. Using in-silico analyses, we found that host genes that contain seed matching sites for their intronic miRNAs yield longer 32UTRs with more polyadenylation sites. Additionally, the distribution of polyadenylation signals differed significantly between these host genes and host genes of miRNAs that do not contain potential miRNA binding sites. We then transferred these in-silico results to a biological example and investigated the relationship between ZFR and its intronic miRNA miR-579 in a U87 cell line model. We found that ZFR is targeted by its intronic miRNA miR-579 and that alternative polyadenylation allows differential targeting. We additionally used bioinformatics analyses and RNA-Seq to evaluate a potential cross-talk between intronic miRNAs and alternative polyadenylation. CPSF2, a gene previously associated with alternative polyadenylation signal recognition, might be linked to intronic miRNA negative feedback by altering polyadenylation signal utilization.

## Introduction

In the recent past, miRNAs have gained significant attention as regulators of the transcriptome. MiRNA genes are found throughout the genome, and about half of them are located in genomic regions that contain protein-coding information. They can be classified as either intergenic or intragenic, and the latter can be subclassified as *exonic* or *intronic* [1]. While some intronic miRNAs may be regulated by their own promoter sequences [2], the expression of the majority of intronic miRNAs depends on transcriptional activation of the host gene: When a protein-coding gene is transcribed into mRNA, this primary transcript also contains the miRNA sequence that may subsequently be processed into a mature miRNA [3]. Consequently, the expression of a miRNA can be coupled to the expression of its host gene. Increasing evidence suggests that this miRNA—host gene relationship is of functional importance: Intronic miRNAs may affect their hosts’ expression or the expression of host-interacting proteins [1]. In both cases, intronic miRNAs were shown to influence the molecular activities of their hosts. Recently, Dill et al. experimentally validated an example of an intronic miRNA targeting its host gene, hence uncovering a direct negative feedback mechanism [4]. Interestingly, the miRNA was processed only after differentiation of the cell, showing that this mechanism was time-dependent. This clearly proved the existence of functional relationships between intronic miRNAs and their host genes. Furthermore, this work identified a first example for regulation of this coupling. However, the described model was limited to cell differentiation processes. So far it remains unclear whether there exist more general mechanisms that may enable control of host gene expression by intronic miRNAs.

Whereas differential processing of the intronic miRNA constitutes one way to control activity of a negative feedback mechanism, modulation of miRNA target-site accessibility may be another option. Many protein-coding genes bear multiple polyadenylation sites in their 32UTRs, enabling the transcription of variable size mRNAs that may or may not contain specific miRNA target sites [5]. Poly(A)-site selection is determined by context and type of polyadenylation signals. In general, canonical polyadenylation signals (“AAUAAA”, “AUUAAA”) are distinguished from non-canonical polyadenylation signals. Several enzymes have been identified that are linked to 3´UTR processing and are commonly referred to as 3´-processing factors, the stoichiometry of which seems to be very influential (for a detailed summary of alternative polyadenylation see [6]). We hypothesized that miRNA target-site accessibility could be modulated by alternative polyadenylation (APA) processes as an additional mechanism of intronic miRNA-driven negative feedback loops. First, we used a bioinformatics approach to investigate, whether APA-motif distribution differs in the 32UTRs of host genes with and without an intronic miRNA seed matching site. We then chose ZFR and its intronic miRNA miR-579 as an example and could show that ZFR is in fact targeted by miR-579. Moreover, we show that there are at least two 32UTR isoforms, one of which contains the miRNA target site while the other doesn’t, proving that alternative polyadenylation is a way for the cell to scale the degree of immediate negative feedback. We also investigated, whether intronic miRNAs targeting their own host gene may interfere with polyadenylation machinery. Using bioinformatics screening for overrepresented potential miRNA targets within the APA machinery, we identified CPSF2 as a potential intronic miRNA target. We show that ZFR targets CPSF2, and that silencing of CPSF2 lead to an increased utilization of canonical polyadenylation signals. These data indicate an interesting link between intronic miRNA feedback and alternative polyadenylation.

## Results and Discussion

### APA regulates the impact of intronic miRNAs on the expression of their host genes

**Fig 1:**
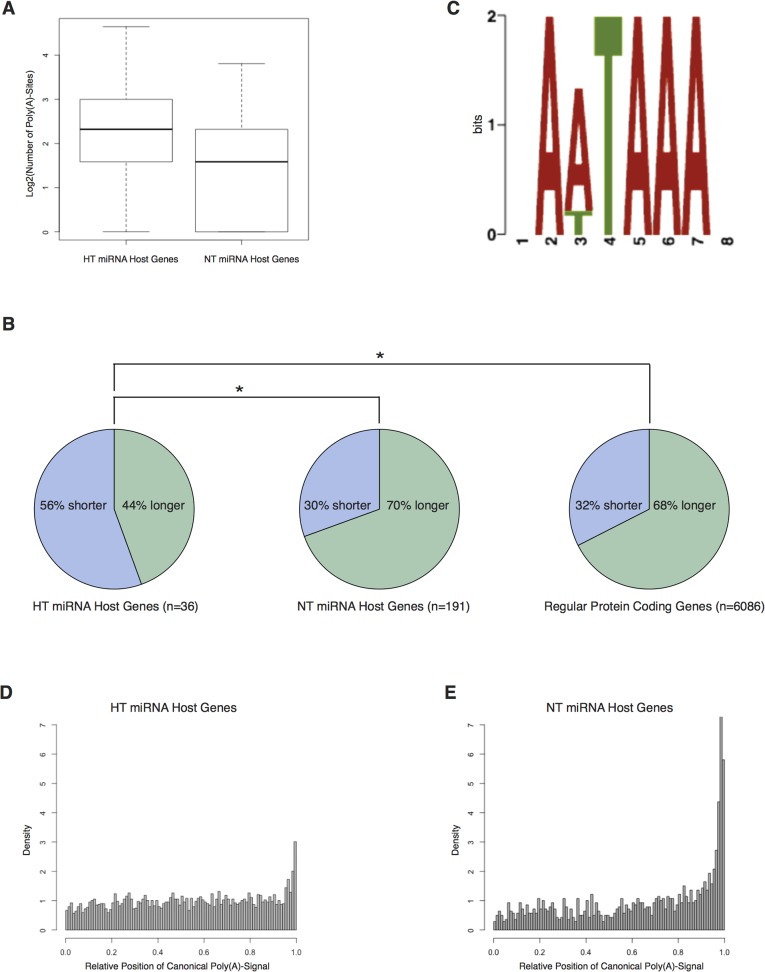
Bioinformatics and biomolecular analyses indicate a role for APA in regulation of negative feedback. A) Comparison of APA-sites for HT miRNA host genes and NT miRNA host genes. B) After CPSF2 silencing HT miRNA host gene UTRs display a different poly(A)-site usage pattern compared to NT miRNA host gene UTRs and regular protein-coding genes’ UTRs. C) The motif discovered in upregulated APA regions after CPSF2 silencing resembles the two canonical polyadenylation sites. D) Distribution of canonical poly(A) signals across the 32UTR of HT miRNA host genes and E) NT miRNA host genes.

To investigate the hypothesis that APA regulates a negative feedback mechanism imposed by miRNAs targeting their own hosts, we first classified intronic miRNAs into host-targeting (HT) miRNAs or non-host-targeting (NT) miRNAs by searching for seed site matches within the respective 32UTR sequences of the host genes. A total of 203 HT miRNAs were located in 168 host genes, with 583 seed site matches. 601 NT miRNAs were located within 351 host genes (see also S1 Fig.). We found that HT miRNA host genes possess longer 32UTR sequences (median = 2553 nt vs median = 1198 nt, P < 2.2E-16) and contain significantly more poly(A) sites than NT miRNA host genes (median = 5 vs median = 3, P = 6.7E-9) (Fig. 1A). Of 583 total seed site matches, 435 HT miRNA-matching seed sites are potentially influenced by APA, affecting 124 of the 168 HT host genes. In summary, our results illustrate that 32UTRs of HT miRNA host genes are longer and contain more APA sites. Long 32UTRs have been shown to preferably occur in genes in which slight expression changes can be detrimental to the cell, thus requiring tight regulation [6]. We then mapped the here analyzed host genes to KEGG (Kyoto Encyclopedia of Genes and Genomes), a database of known biological pathways. We found that many of the here analyzed host genes are linked to signal transduction pathways (S1 Table), thus representing a group of genes in which tight expression control is vital. Furthermore it has been shown that shortening of 32UTRs by APA is a highly effective method to escape regulatory control [7, 8]. Thus, our findings point to a potential regulation of HT miRNA host genes by APA. Based on previous publications [4,7], it is tempting to speculate that differential miRNA maturation, as described by Dill and colleagues, could be primarily used for developmental regulation, while APA might be a primary mechanism in short-term processes, such as immunoactivation [7].

### ZFR is targeted and differentially regulated by its intronic miRNA hsa-miR-579

After evaluation of binding probabilities and UTR-lengths of potential candidate host genes harboring intronic miRNAs with a seed-matching motif in their 32UTR, *ZFR* (Zink-finger recombinase) was chosen as the example molecule for further evaluation.

**Fig 2:**
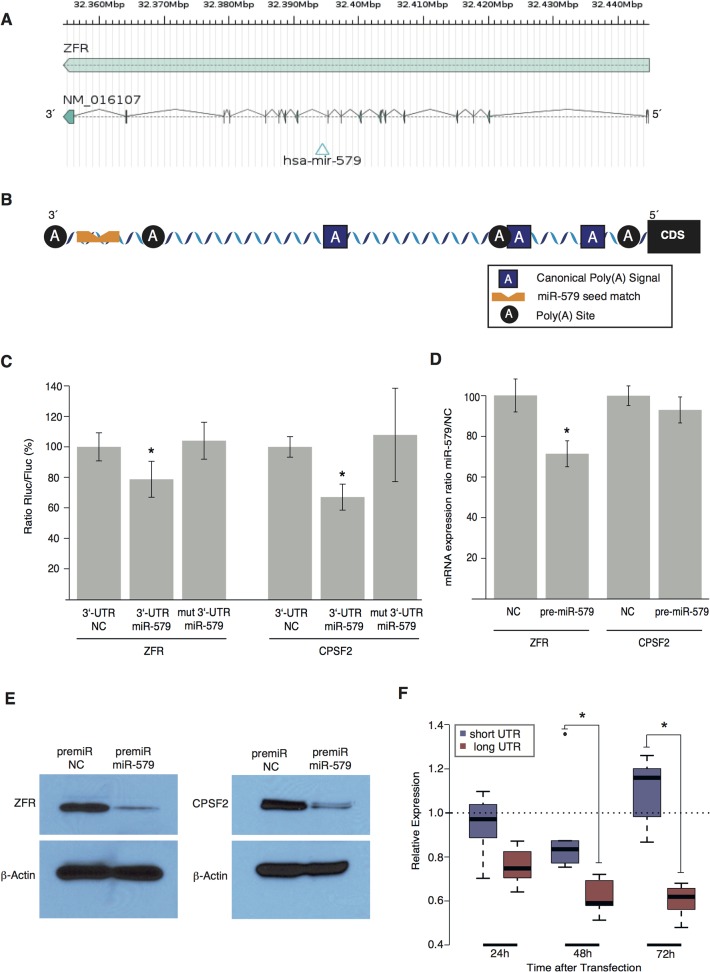
miR-579 targets its host, ZFR, and the APA associated gene CPSF2. A) Schematic diagram of the *ZFR* gene. B) Schematic diagram of the ZFR 32UTR including polyadenylation sites and the seed matching site for miR-579. C) U87 cells were co-transfected with reporter constructs containing wildtype ZFR-32UTR or ZFR-32UTR lacking the miR-579 binding site (mut 32UTR) along with pre-miR-579 or negative control (NC). Results are expressed as Rluc/Fluc ratio relative to NC (mean ± 95% CI; n = 6; *, p < 0.05). D) In U87 cells transiently transfected with scrambled control or pre-miR-579, ZFR and CPSF2 mRNA expression was analyzed by quantitative RT-PCR. Values are mean ± 95% CI; n = 5; *, p < 0.05. E) Western blot analysis of the same samples using specific antibodies as indicated (β-Actin served as loading control; one representative experiment of three is shown). F) In U87 cells, expression changes of the long (miRNA binding site containing; red) and short (without miRNA binding site; blue) alternatively polyadenylated UTRs after transfection with pre-miR-579 or with scrambled control was determined by quantitative RT-PCR. Values are shown as miR-579 transfection relative to scrambled control (n = 5; *, p < 0.05).

*ZFR* encodes a three zinc-finger protein [9] with a total length of 90,389 base pairs, 19 intronic regions and a 32UTR length of 1,409 nucleotides (Fig. 2A). It hosts the human-specific miRNA gene hsa-mir-579 in intron 11 (intron length: 4,722 bp, distance to the upstream exon: 684 bp), which appears to be co-expressed with its host gene, as there is no bioinformatic evidence of an individual promoter region for this miRNA. Even though not well characterized, recent literature suggests an important role for ZFR in neuron development [10]. It contains a seed site for hsa-miR-579 at position-chr5:32,354,558–32,354,564 and, according to our database, APA sites at positions chr5:32,354,730, chr5:32,355,524, and chr5:32,355,823 (Fig. 2B). Importantly, only the longest UTR isoform harbors the binding site for hsa-miR-579 at nucleotide position 1301 after the CDS. Canonical polyadenylation signal motifs appear at 135, 314 (AUUAAA), and 738 (AAUAAA) nucleotides. These isoforms were validated using 32RACE with subsequent sequencing (S2 Fig.).

To experimentally validate the direct binding and targeting of hsa-miR-579 to its host ZFR, we subcloned its 32UTR into the MCS of the psiCheck-2 vector. This vector contains both *Renilla reniformis* luciferase (Rluc) and *Photinus pyralis* (Firefly) luciferase (Fluc) on a single plasmid with the MCS located downstream of the *Renilla* encoding region. The reporter vectors were co-transfected with pre-miR-579 (or with scrambled control) and Rluc/Fluc ratios were calculated. Luciferase activity was significantly repressed (inhibition by 21.3 ± 11.9%); this effect could be counteracted by introducing a single-nucleotide mutation in the seed matching sequence (Fig. 2C). After pre-miR-579 transfection of U87 cells, a decrease of mRNA levels of ZFR (29%) was observed (Fig. 2D). Western blotting confirmed a significant protein reduction (Fig. 2E). These data show that miR-579 not only targets its host ZFR, but due to the position of the polyadenylation sites, this interaction might be differentially controlled. To investigate this assumption, we transfected pre-miR-579 into U87 cells and measured the expression of both the short and the long, miR-579-seed site match-containing UTR of the ZFR transcript during a time period extending from 24 h to 72 h after transfection. As shown in Fig. 2F, the abundance of the long UTR decreases over time (median expression after 72h was decreased by 38% [range 32%–52% decrease] compared to normal control), while the short variant is not affected (median decrease 16% [range 26% decrease—13% increase]).

APA may thus be a mechanism for the cell to selectively enable and disable direct negative feedback of host genes by their intronic miRNAs.

### HT miRNAs influence the host gene’s accessibility by targeting the APA machinery

Given the potential influence of APA on miRNA targeting we hypothesized that some miRNAs themselves might actually influence the decision of which polyadenylation site is chosen. One such mechanism would be the targeting of components of the APA machinery, which, via a change of stoichiometry of APA components, might influence the target accessibility of their host genes. We thus analyzed a set of 11 genes that have recently been associated with polyadenylation signal recognition (Table 1) [11]. 32UTR regions were searched in-silico for miRNA seed site matches. Generally, all investigated genes exhibited seed site matches for a larger fraction of HT miRNAs when compared to NT miRNAs or to intergenic miRNAs. Among these genes, CPSF2, a gene linked to the recognition of polyadenylation signals [12, 13], yielded the most significant difference in potential binding sites. Since CPSF2’s 32UTR contains a seed-matching motif for miR-579 at 168 bp after the CDS, we first investigated, if CPSF2 is a target of miR-579. Using the aforementioned reporter vector assay, luciferase activity was significantly repressed (inhibition of 33.0 ± 8.5%) and recovered by introduction of a single-point mutation (Fig. 2C). While CPSF2 mRNA levels were unaffected after miR-579 transfection (Fig. 2D), western blotting revealed a significant reduction in CPSF2 protein abundance (Fig. 2E). These results could be interpreted that either miR-579 regulates CPSF2 expression via translational repression or that mRNA changes may occur outside of the analyzed time window. To further elucidate the role of CPSF2 in the context of alternative polyadenylation, U87 cells were transfected with specific siRNAs against CPSF2 resulting in a reduction of CPSF2 mRNA of more than 90%. Subsequently, cells’ transcriptome was sequenced using an AB-SOLiD platform. First, potential polyadenylation sites were identified and the reads were mapped to the respective polyadenylation areas. Genes were then filtered for sequencing depth and significant changes in 32UTR poly(A) region usage (at least one significant increased and at least one significant decreased poly(A) region per 32UTR), a total of 6313 genes were subject to further analysis (36 HT miRNA host genes, 191 NT miRNA host genes, 6086 regular protein coding genes). On average, the mapped reads-count for poly(A)-regions that were more distant from the CDS increased, whereas the mapped reads-count for closer regions decreased after CPSF2-silencing, suggesting an elongation of the 32UTR. Surprisingly, the majority of HT miRNA host genes displayed a significant opposite effect: 32UTRs were shortened (Fig. 1B, Table 2). To find an explanation for these observations, we analyzed the sequence-blocks that most significantly gained read counts using the MEME web tool for overrepresented motifs [14]. The most significant motif found resembles the consensus sequence of the two known canonical polyadenylation signals (Fig. 1C), strongly suggesting a role of CPSF2 in utilization of non-canonical polyadenylation signals. As it is known, that canonical polyadenylation signals tend to be located near the outmost 32 region of a UTR [15], the supposed general tendency towards longer 32UTRs could be well explained by a model where CPSF2 is responsible for the recognition of non-canonical poly(A)-signals. As HT miRNA host genes did not follow that general rule, we compared distributions of the relative position of canonical polyadenylation signals within HT host gene UTRs and NT host gene UTRs. Indeed, distribution patterns for canonical poly(A)-signals in HT miRNA host genes significantly differed from NT miRNA host genes (median = 0.55 vs median = 0.73, p < 2.2E-16): While poly(A)-signals in NT miRNA host genes accumulate at the 32 end of the UTR, thus resembling the distribution of the majority of protein-coding genes, they tend to be more evenly distributed in HT miRNA host genes (Fig. 1D and1E). In fact, 473 of the 583 HT seed matching motifs were preceded by a canonical poly(A) signal, offering an explanation why more than half of the significantly affected HT host gene UTRs showed a pattern of utilization of more proximal poly(A)-sites.

**Table 1:** Identification of APA genes preferentially targeted by HT miRNAs.

Gene Symbol	HT versus NT miRNAs	q-value	HT versus intergenic miRNAs	q-value
CSTF1	28 (14%) vs 61 (10%)	0.371	28 (14%) vs 100 (10%)	0.333
CSTF2	76 (37%) vs 172 (29%)	0.171	76 (37%) vs 288 (29%)	0.171
CSTF3	23 (11%) vs 63 (11%)	0.827	23 (11%) vs 135 (13%)	0.495
CPSF1	7 (3%) vs 6 (1%)	0.171	7 (3%) vs 31 (3%)	0.827
CPSF2	79 (38%) vs 158 (27%)	0.021	79 (38%) vs 258 (26%)	0.01
CPSF3	6 (3%) vs 9 (2%)	0.371	6 (3%) vs 14 (1%)	0.333
CPSF4	28 (14%) vs 62 (10%)	0.371	28 (14%) vs 105 (10%)	0.371
NUDT21	81 (39%) vs 201 (34%)	0.371	81 (39%) vs 353 (35%)	0.371
CPSF6	136 (66%) vs 365 (61%)	0.371	136 (66%) vs 618 (61%)	0.371
CPSF7	138 (67%) vs 380 (64%)	0.55	138 (67%) vs 631 (63%)	0.371
FIP1L1	19 (9%) vs 30 (5%)	0.171	19 (9%) vs 55 (5%)	0.171

**Table 2:** HT miRNA host genes with significant 3´UTR changes after CPSF2-silencing.

host gene symbol	miRNA symbol	HT miRNA	3´UTR change
CHM	hsa-miR-361-5p	yes	shorter UTR
CHM	hsa-miR-361-3p	no	shorter UTR
DKC1	hsa-miR-644b-5p	no	shorter UTR
DKC1	hsa-miR-644b-3p	yes	shorter UTR
GPC1	hsa-miR-149-5p	yes	shorter UTR
GPC1	hsa-miR-149-3p	yes	shorter UTR
HNRNPK	hsa-miR-7-5p	no	shorter UTR
HNRNPK	hsa-miR-7-1-3p	yes	shorter UTR
TNPO1	hsa-miR-4804-5p	no	shorter UTR
TNPO1	hsa-miR-4804-3p	yes	shorter UTR
LPP	hsa-miR-28-5p	no	shorter UTR
LPP	hsa-miR-28-3p	yes	shorter UTR
MLLT6	hsa-miR-4726-5p	yes	shorter UTR
MLLT6	hsa-miR-4726-3p	no	shorter UTR
NHS	hsa-miR-4768-3p	no	shorter UTR
NHS	hsa-miR-4768-5p	yes	shorter UTR
SREBF1	hsa-miR-33b-5p	yes	shorter UTR
SREBF1	hsa-miR-33b-3p	no	shorter UTR
PPFIA1	hsa-miR-548k	yes	shorter UTR
ALDH4A1	hsa-miR-4695-5p	yes	shorter UTR
ALDH4A1	hsa-miR-1290	no	shorter UTR
ALDH4A1	hsa-miR-4695-3p	yes	shorter UTR
CTDSP2	hsa-miR-26a-5p	yes	shorter UTR
CTDSP2	hsa-miR-26a-2-3p	no	shorter UTR
COPZ1	hsa-miR-148b-3p	yes	shorter UTR
COPZ1	hsa-miR-148b-5p	yes	shorter UTR
DPY19L1	hsa-miR-548n	yes	shorter UTR
ZFR	hsa-miR-579	yes	shorter UTR
GALNT7	hsa-miR-548t-5p	yes	shorter UTR
GALNT7	hsa-miR-548t-3p	no	shorter UTR
RBM47	hsa-miR-4802-3p	yes	shorter UTR
RBM47	hsa-miR-4802-5p	no	shorter UTR
GALNT10	hsa-miR-1294	yes	shorter UTR
C9orf3	hsa-miR-23b-3p	no	shorter UTR
C9orf3	hsa-miR-24-3p	no	shorter UTR
C9orf3	hsa-miR-24-1-5p	yes	shorter UTR
C9orf3	hsa-miR-27b-5p	no	shorter UTR
C9orf3	hsa-miR-2278	yes	shorter UTR
C9orf3	hsa-miR-23b-5p	no	shorter UTR
C9orf3	hsa-miR-27b-3p	no	shorter UTR
LASS6	hsa-miR-4774-3p	yes	shorter UTR
LASS6	hsa-miR-4774-5p	no	shorter UTR
ADCY6	hsa-miR-4701-3p	yes	longer UTR
ADCY6	hsa-miR-4701-5p	no	longer UTR
CD58	hsa-miR-548ac	yes	longer UTR
NFYC	hsa-miR-30c-5p	no	longer UTR
NFYC	hsa-miR-30c-1-3p	yes	longer UTR
NFYC	hsa-miR-30e-3p	no	longer UTR
NFYC	hsa-miR-30e-5p	no	longer UTR
SCP2	hsa-miR-1273g-3p	yes	longer UTR
SCP2	hsa-miR-1273g-5p	no	longer UTR
SCP2	hsa-miR-5095	no	longer UTR
SCP2	hsa-miR-1273f	yes	longer UTR
ZRANB2	hsa-miR-186-5p	yes	longer UTR
ZRANB2	hsa-miR-186-3p	yes	longer UTR
BRE	hsa-miR-4263	yes	longer UTR
ARHGEF11	hsa-miR-765	yes	longer UTR
AP3S2	hsa-miR-5094	yes	longer UTR
AP3S2	hsa-miR-5009-3p	yes	longer UTR
AP3S2	hsa-miR-5009-5p	yes	longer UTR
IGF2BP2	hsa-miR-548aq-3p	yes	longer UTR
IGF2BP2	hsa-miR-548aq-5p	no	longer UTR
HBS1L	hsa-miR-3662	yes	longer UTR
C9orf5	hsa-miR-32-3p	yes	longer UTR
C9orf5	hsa-miR-32-5p	no	longer UTR
PITPNC1	hsa-miR-548aa	yes	longer UTR
ATAD2	hsa-miR-548d-5p	yes	longer UTR
ATAD2	hsa-miR-548d-3p	yes	longer UTR
FBXW7	hsa-miR-3140-5p	no	longer UTR
FBXW7	hsa-miR-3140-3p	yes	longer UTR
NMNAT1	hsa-miR-5697	yes	longer UTR
RASSF3	hsa-miR-548c-5p	no	longer UTR
RASSF3	hsa-miR-548c-3p	yes	longer UTR

We thus identified CPSF2 as a molecule that is potentially targeted by several intronic miRNAs. When silenced, polyadenylation seemed to be biased towards recognition of canonical poly(A)-signals, suggesting 32UTR elongation for the majority of genes, and 32UTR shortening in a significant fraction of HT host genes.

**Fig 3:**
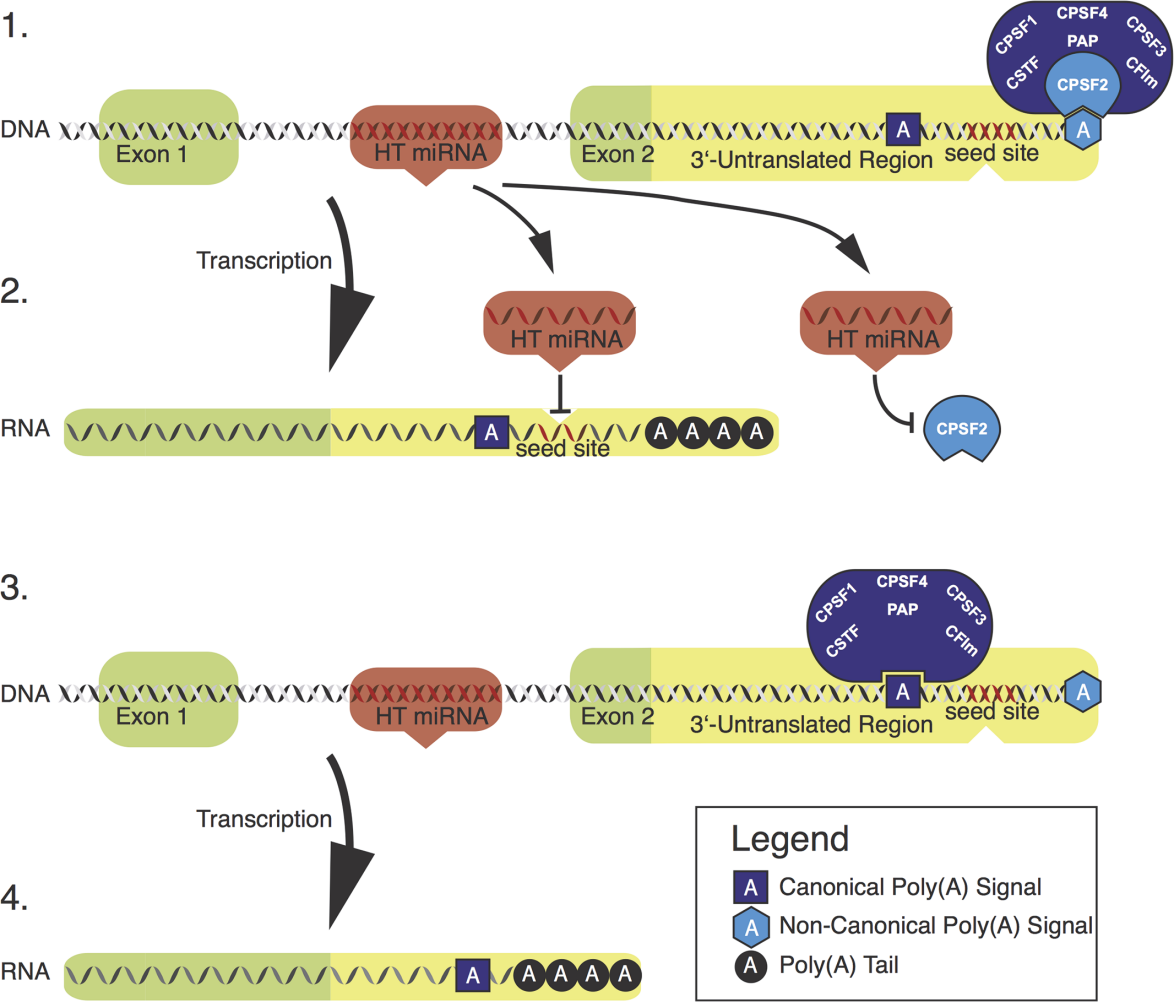
Model of intronic negative feedback regulation. After coexpression of miRNA and host gene, the miRNA directly regulates its host gene as well as CPSF2. After removal of CPSF2 the polyadenylation-complex is biased towards recognition of canonical sites. In the next transcription cycle, the canonical site that precedes the miRNA binding site is utilized. Hence, regulation of the host gene by its intronic miRNA is disabled.

**Fig 4:**
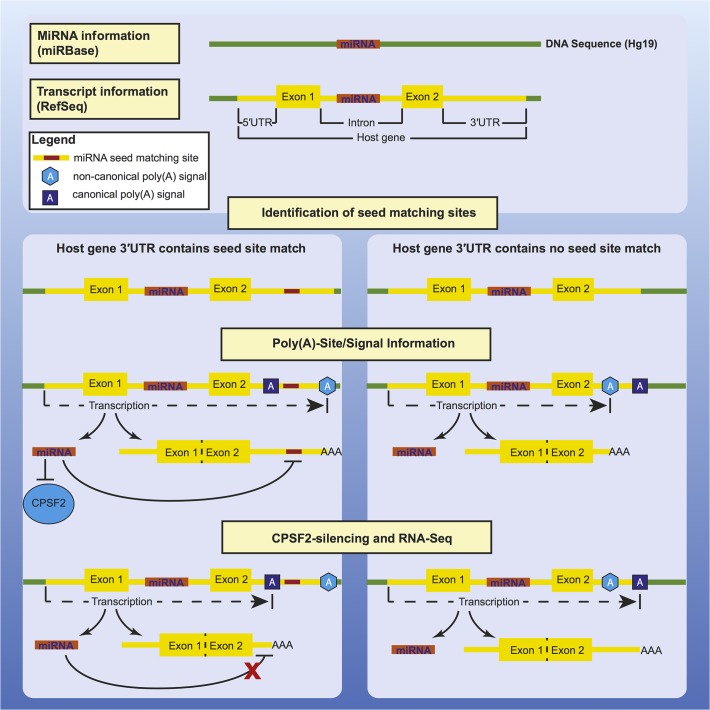
Summary.

These findings may point to a new model for regulation of miRNA host gene expression via alternative polyadenylation (Figs. 3 and 4): After co-expression of host gene and its intronic miRNA, the miRNA is able to regulate its host gene by binding to the 32UTR. Simultaneously, the miRNA targets CPSF2, thereby changing the stoichiometry of polyadenylation factors. Subsequently, canonical poly(A)-signals are preferred over non-canonical signals leading to a shortening of the host gene UTR with consecutive loss of the seed site match. This leads to a decoupling of the negative feedback circuitry.

### Conclusions

The persistent transcriptional coupling of a miRNA with its host that is also its target would per se not be very useful. Thus, mechanisms allowing a differential regulation need to exist. While previous authors described differential intronic miRNA processing as one mechanism [4], we investigated the relationship between *ZFR* and its intronic miRNA *hsa-mir-579* and found another possibility of regulation. We could show that miR-579 targets its host ZFR, and that via APA two ZFR transcripts exist, one that is targeted by its intronic miRNA, and another one that is not. As an addition, we provide evidence that APA in turn might be influenced by intronic miRNAs through interfering with the expression of CPSF2, suggesting that at least some intronic miRNAs might even be able to turn negative feedback off themselves.

It is tempting to speculate that differential miRNA processing is a technique primarily employed during organism development and cell differentiation, while alternative polyadenylation appears to be a mechanism for responding to environmental factors, such as described by Sandberg and colleagues.

As an abstraction of our results, we depict a hypothetical model of intronic miRNA feedback regulation in Fig. 4: After expression of the host gene and its intronic miRNA, the miRNA is able to regulate its host gene by binding to the 3´UTR. Simultaneously, the miRNA targets the 3´UTR-processing factor CPSF2, thereby changing the stoichiometry of polyadenylation factors. Subsequently canonical poly(A)-signals are preferred over non-canonical signals, leading to a shortening of host gene UTRs of these miRNAs with subsequent loss of the seed site match. This leads to decoupling of the negative feedback circuitry.

Due to the nature of miRNAs as fine-tuners of gene expression, it is unlikely that expressional changes of a single miRNA in vivo are enough to sufficiently change CPSF2 expression. Additional miRNAs and further regulatory mechanisms are needed to exert the proposed effect.

Even though reality is doubtless more complex than appreciated in the current work, our results may unveil an important piece in the understanding of miRNA based negative feedback circuitries.

## Methods

### Datasources

MySQL version 5.0 was used on a dual core server running Ubuntu Linux. The database was accessed using Python 2.7 with the Pygr and MySQLdb libraries. MiRNA seed complementary sites were identified by searching 32UTRs for a complete complementary match of nucleotides 2–8 of the mature miRNA sequence or a match of nucleotides 2–7 followed by an adenine (‘A’). The human reference genome sequence (hg19/GRCh37), gene transcription annotation information and human transcriptome data from the Reference Sequence Project (RefSeq; Release #49) [16], were downloaded from the UCSC Genome Browser [17, 18] and retrieved from the NCBI’s ftp-server. miRNA genomic coordinates, seed sequences, and family information were derived from miRBase version 18 [19, 20]. The database was constructed as previously described [21].

### Identification of APA Sites

Three different datasources were integrated for the analysis. First, we mapped all expressed sequence tag (EST) sequences to the human reference genome using a previously described protocol [22, 23]. Only sequences with an adenine stretch of more than 10 untemplated nucleotides in the 3´ extremity were selected. Internally primed ESTs were removed and chimeras and paralogs were controlled for. Second, APA site data across five human tissues derived from PolyA-Seq were integrated into this data source [5]. Third, RNA-Seq data (see below) were used to identify potential APA sites. Color code reads were required to contain at least two untemplated “0”s as well as at least two reads for the same site of different mapping length. APA sites within a distance of 40 nucleotides were subsumed into one site. Only sites within the longest annotated RefSeq transcript were considered.

### Poly(A)+ libraries construction and sequencing

To prepare Poly(A)+ libraries, we started with 500 ng Poly(A)+ RNA from each sample. The RNA was fragmented using RNAse III, followed by ligation of SOLiD adaptors, reverse transcription, and size selection for subsequent amplification, according to the manufacturers’ instructions (Life Technologies). After assessing the amplified DNA for yield and size distribution on the Bioanalyzer instrument (Agilent), libraries were submitted to emulsion PCR followed by sequencing on a SOLiD4 System.

### Bioinformatics analysis of RNA-Seq data

A total of ∼ 50 million color code reads for CPSF2-silenced cells (study data) and ∼ 100 million color code reads for cells transfected with a non-functional pre-miRNA (control data) were analyzed. Data were deposited at [SRA-ACC:SRP053217]. All generated reads were mapped against the human reference genome using the genome mapping pipeline from Bioscope (standard parameters). All alignments were converted to BAM format and only alignments with a quality score ≥ 20 (guaranteeing an alignment error-rate of at most 1% and a unique genome match per read) were selected. These mapped reads were crossed with gene annotation and APA information, and read counts for each poly(A) region were calculated. Statistical significance of read count changes was assessed using the binomial test. A gene’s 32UTR was considered prolonged in the study group when the median index of significantly upregulated poly(A)-blocks was greater than the median index of significantly downregulated poly(A)-blocks and shortened otherwise. Only genes that contained both significantly up- and downregulated poly(A)-blocks were considered. The MEME tool was used with standard parameters (motif occurrences per sequence: 0 or 1, motif-width: 6–20, number of motifs: 0–5) on the 292 most significantly upregulated poly(A)-region sequences as positive and 89 most significantly downregulated poly(A)-region sequences as negative controls [12]. Of each of these regions, 40 nucleotides upstream of the poly(A)-site were used.

### Statistical analysis

We performed all statistical calculations using the statistical programming software R or the Stats-library from the python scientific computing project SciPy [24]. The Mann-Whitney-U test was used for the assessment of statistical significance of differences in 32UTR lengths and number of APA sites between intronic host-targeting (HT) miRNAs and intronic non-host-targeting (NT) miRNAs. We applied the Fisher’s exact test for identification of genes preferentially targeted by HT miRNAs. Correction for multiple hypothesis testing done using the Benjamini-Hochberg algorithm where appropriate. We followed the seed matching motif algorithm of popular target prediction tools and required either a base-complementary match of nucleotides 2–8, or Mapping of HT miRNAs to the Kyoto Encyclopedia of Genes and Gene Products (KEGG) and to the Gene Ontology biological function was carried out using R’s bioconductor packages GOstats, KEGG.db, GO.db, org.Hs.eg.db, and Cytoscape in combination with the Bingo plugin [25–29]. qPCR and Luciferase measurements were normalized across the three replicates of the normal control. Statistical significance was assessed using the Mann-Whitney-U test. Throughout the whole manuscript a significance level of < 0.05 was used.

### Cell culture

U87 cells (American Type Culture Collection) were grown at 37°C and 5% CO2 in Dulbecco’s modified Eagle medium (Lonza) supplemented with 10% heat-inactivated FCS, 1% penicillin/streptomycin/glutamine (v/v) and 1% NEAA.

### Transfection and reporter gene assay

Cell transfection experiments were performed using the Neon Transfection System (Invitrogen). U87 cells were transiently transfected with ON-TARGETplus SMARTpool siRNA against CPSF2 or negative control (Dharmacon) at final concentrations of 50 nM. Cells were harvested 96 hours later. The psiCheck-2 Dual-Luciferase Vector (Promega) was used for the generation of reporter constructs (for details see S1 File). U87 cells were co-transfected with 1 μg psiCheck-2 reporter vector containing ZFR or CPSF2 32UTR variants with pre-miR miRNA precursor molecules (Ambion) at final concentrations of 50 nM. After 40 hours, luciferase activity was analyzed using the Dual-Glo Luciferase Assay System (Promega) and Renilla luciferase activities were normalized to Firefly luciferase activities. All data resulted from five or more independent experiments.

### RNA isolation and synthesis of cDNA

Total RNA was isolated using the RNAqueos Kit (Ambion) with subsequent DNase treatment (Turbo DNA-free Kit, Ambion). RNA quantity was determined using the NanoDrop ND-1000 spectrophotometer (Peqlab). cDNA was synthesized from 1 μg of total RNA using the SuperScriptIII First Strand Synthesis System (Invitrogen) and random hexamers. For quantification of ZFR long and short UTRs, a primer-specific reverse transcription was performed using the poly(A)-Linker listed in S1 File.

### PCR experiments

Quantitative real-time PCR was performed on a Light Cycler 480 (Roche Diagnostics) using Roche´s UPL probes. For quantification of ZFR long and short UTRs, a reverse primer specifically annealing on the poly(A)-linker in combination with specific forward primers was used for qPCR together with Roche's SYBR Green. Cycling conditions were 45 cycles of 95°C for 10 s, 60°C for 10 s, and 72°C for 15 s. Specificity was verified by melting point analysis. In all cases, reference gene normalization to SDHA and TBP as previously described [30]. All qPCR primers are listed in S1 File. 32RLM-RACE was performed using the FirstChoice RLM-RACE Kit (Ambion) and the primers listed in S1 File. PCR products were subcloned into the StrataClone Blunt Vectoramp/kan (Stratagene) and sequenced.

### Western blot analysis

Western blotting was performed with 30 μg of total protein extract and antibodies against ZFR or CPSF2 (both: Abcam). Mouse monoclonal anti-β-actin antibody served as a loading control. Immunoreactive bands were detected using goat anti-rabbit or goat anti-mouse HRP conjugates (Cell Signaling Technologies).

## Supporting Information

S1 Fig Classification of miRNAs into intronic, exonic, and intergenic miRNAs. (TIFF)

S2 Fig 3´RACE. (TIFF)

S1 File Extended information on Luciferase vector construction and primer sequences. (PDF)

S1 Table Mapping of host-targeting intronic miRNA host genes to the KEGG ontology pathways. (XLS)

## References

[1] Hinske LCG, Galante PAF, Kuo WP, Ohno-Machado L. A potential role for intragenic miRNAs on their hosts’ interactome. BMC genomics 2010;11:533 10.1186/1471-2164-11-533 20920310 PMC3091682

[2] Monteys AM, Spengler RM, Wan J, Tecedor L, Lennox KA, Xing Y, et al Structure and activity of putative intronic miRNA promoters. RNA (New York, NY) 2010;16:495–505. 10.1261/rna.1731910 20075166 PMC2822915

[3] Kim Y-K, Kim VN. Processing of intronic microRNAs. The EMBO Journal 2007;26:775–783. 17255951 10.1038/sj.emboj.7601512 PMC1794378

[4] Dill H, Linder B, Fehr A, Fischer U. Intronic miR-26b controls neuronal differentiation by repressing its host transcript, ctdsp2. Genes & development 2012;26:25–30. 22215807 10.1101/gad.177774.111 PMC3258962

[5] Derti A, Garrett-Engele P, Macisaac KD, Stevens RC, Sriram S, Chen R, et al. A quantitative atlas of polyadenylation in five mammals. Genome Research 2012. 10.1101/gr.132563.111 PMC3371698 22454233

[6] Di Giammartino DC, Nishida K, Manley JL Mechanisms and consequences of alternative polyadenylation. Molecular cell 2011;43:853–866. 10.1016/j.molcel.2011.08.017 21925375 PMC3194005

[7] Sandberg R, Neilson JR, Sarma A, Sharp PA, Burge CB. Proliferating cells express mRNAs with shortened 3’ untranslated regions and fewer microRNA target sites. Science (New York, NY) 2008;320:1643–1647. 10.1126/science.1155390 18566288 PMC2587246

[8] Mayr C, Bartel DP. Widespread shortening of 3’UTRs by alternative cleavage and polyadenylation activates oncogenes in cancer cells. Cell 2009;138:673–684. 10.1016/j.cell.2009.06.016 19703394 PMC2819821

[9] Elvira G, Massie B, DesGroseillers L. The zinc-finger protein ZFR is critical for Staufen 2 isoform specific nucleocytoplasmic shuttling in neurons. Journal of neurochemistry 2006;96:105–117. 16277607 10.1111/j.1471-4159.2005.03523.x

[10] Barber JCK, Huang S, Bateman MS, Collins AL. Transmitted deletions of medial 5p and learning difficulties; Does the cadherin cluster only become penetrant when flanking genes are deleted? American journal of medical genetics Part A 2011. 10.1002/ajmg.a.34241 21965044

[11] Martin G, Gruber AR, Keller W, Zavolan M. Genome-wide Analysis of Pre-mRNA 3’End Processing Reveals a Decisive Role of Human Cleavage Factor I in the Regulation of 3&amp;prime; UTR Length. CellReports 2012;1:753–763. 10.1016/j.celrep.2012.05.003 22813749

[12] Kolev NG, Yario TA, Benson E, Steitz JA. Conserved motifs in both CPSF73 and CPSF100 are required to assemble the active endonuclease for histone mRNA 3&apos;-end maturation. EMBO reports 2008;9:1013–1018. 10.1038/embor.2008.146 18688255 PMC2572124

[13] Herr AJ, Molnàr A, Jones A, Baulcombe DC. Defective RNA processing enhances RNA silencing and influences flowering of Arabidopsis. Proceedings of the National Academy of Sciences of the United States of America 2006;103:14994–15001. 17008405 10.1073/pnas.0606536103 PMC1581427

[14] Bailey TL, Williams N, Misleh C, Li WW. MEME: discovering and analyzing DNA and protein sequence motifs. Nucleic Acids Research 2006;34:W369–373. 16845028 10.1093/nar/gkl198 PMC1538909

[15] Beaudoing E, Freier S, Wyatt JR, Claverie JM, Gautheret D. Patterns of variant polyadenylation signal usage in human genes. Genome Research 2000;10:1001–1010. 10899149 10.1101/gr.10.7.1001 PMC310884

[16] Pruitt KD, Tatusova T, Maglott DR. NCBI Reference Sequence (RefSeq): a curated non-redundant sequence database of genomes, transcripts and proteins. Nucleic Acids Research 2005;33:D501–504. 15608248 10.1093/nar/gki025 PMC539979

[17] Karolchik D, Hinrichs AS, Kent WJ. The UCSC Genome Browser. In: Current protocols in bioinformatics 2009, Edited by Andreas D Baxevanis [et al] Chapter 1:Unit1.4. 10.1002/0471250953.bi0104s28 PMC2834533 19957273

[18] Mangan ME, Williams JM, Kuhn RM, Lathe WC. The UCSC genome browser: what every molecular biologist should know. In: Current protocols in molecular biology 2009, Edited by Frederick M Ausubel [et al] Chapter 19:Unit19.19. 10.1002/0471142727.mb1909s88 PMC4996636 19816931

[19] Griffiths-Jones S, Saini HK, Van Dongen S, Enright AJ. miRBase: tools for microRNA genomics. Nucleic Acids Research 2008;36:D154–158. 17991681 10.1093/nar/gkm952 PMC2238936

[20] Griffiths-Jones S. The microRNA Registry. Nucleic Acids Research 2004;32:D109–111. 14681370 10.1093/nar/gkh023 PMC308757

[21] Hinske LC, Heyn J, Galante PAF, Ohno-Machado L, Kreth S. Setting Up an Intronic miRNA Database. Methods in molecular biology (Clifton, NJ) 2013;936:69–76. 23007499 10.1007/978-1-62703-083-0_5

[22] Galante PAF, Parmigiani RB, Zhao Q, Caballero OL, de Souza JE, Navarro FCP, et al Distinct patterns of somatic alterations in a lymphoblastoid and a tumor genome derived from the same individual. Nucleic Acids Research 2011;39:6056–6068. 10.1093/nar/gkr221 21493686 PMC3152357

[23] da Cunha JPC, Galante PAF, de Souza JE, de Souza RF, Carvalho PM, Ohara DT, et al Bioinformatics construction of the human cell surfaceome. Proceedings of the National Academy of Sciences of the United States of America 2009;106:16752–16757. 10.1073/pnas.0907939106 19805368 PMC2757864

[24] Oliphant TE. Python for Scientific Computing. Computing in Science & Engineering 2007;9:10–20.

[25] Kanehisa M, Goto S. KEGG: kyoto encyclopedia of genes and genomes. Nucleic Acids Research 2000;28:27–30. 10592173 10.1093/nar/28.1.27 PMC102409

[26] Falcon S, Gentleman R. Using GOstats to test gene lists for GO term association. Bioinformatics (Oxford, England) 2007;23:257–258. 17098774 10.1093/bioinformatics/btl567

[27] Maere S, Heymans K, Kuiper M. BiNGO: a Cytoscape plugin to assess overrepresentation of gene ontology categories in biological networks. Bioinformatics (Oxford, England) 2005;21:3448–3449. 15972284 10.1093/bioinformatics/bti551

[28] Ashburner M, Ball CA, Blake JA, Botstein D, Butler H, Cherry JM, et al Gene ontology: tool for the unification of biology. The Gene Ontology Consortium. Nature genetics 2000;25:25–29. 10802651 10.1038/75556 PMC3037419

[29] Gentleman RC, Carey VJ, Bates DM, Bolstad B, Dettling M, Dudoit S, et al Bioconductor: open software development for computational biology and bioinformatics. Genome Biology 2004;5:R80 15461798 10.1186/gb-2004-5-10-r80 PMC545600

[30] Kreth S, Heyn J, Grau S, Kretzschmar HA, Egensperger R, Kreth FW. Identification of valid endogenous control genes for determining gene expression in human glioma. Neuro-oncology 2010;12:570–579. 10.1093/neuonc/nop072 20511187 PMC2940642

