# Supplementary figures and images for: Alternative Polyadenylation Allows Differential Negative Feedback of Human miRNA miR-579 on Its Host Gene ZFR
Source: PLoS One. 2015 Mar 23;10(3):e0121507. doi: 10.1371/journal.pone.0121507 (PMC4370670; doi:10.1371/journal.pone.0121507)

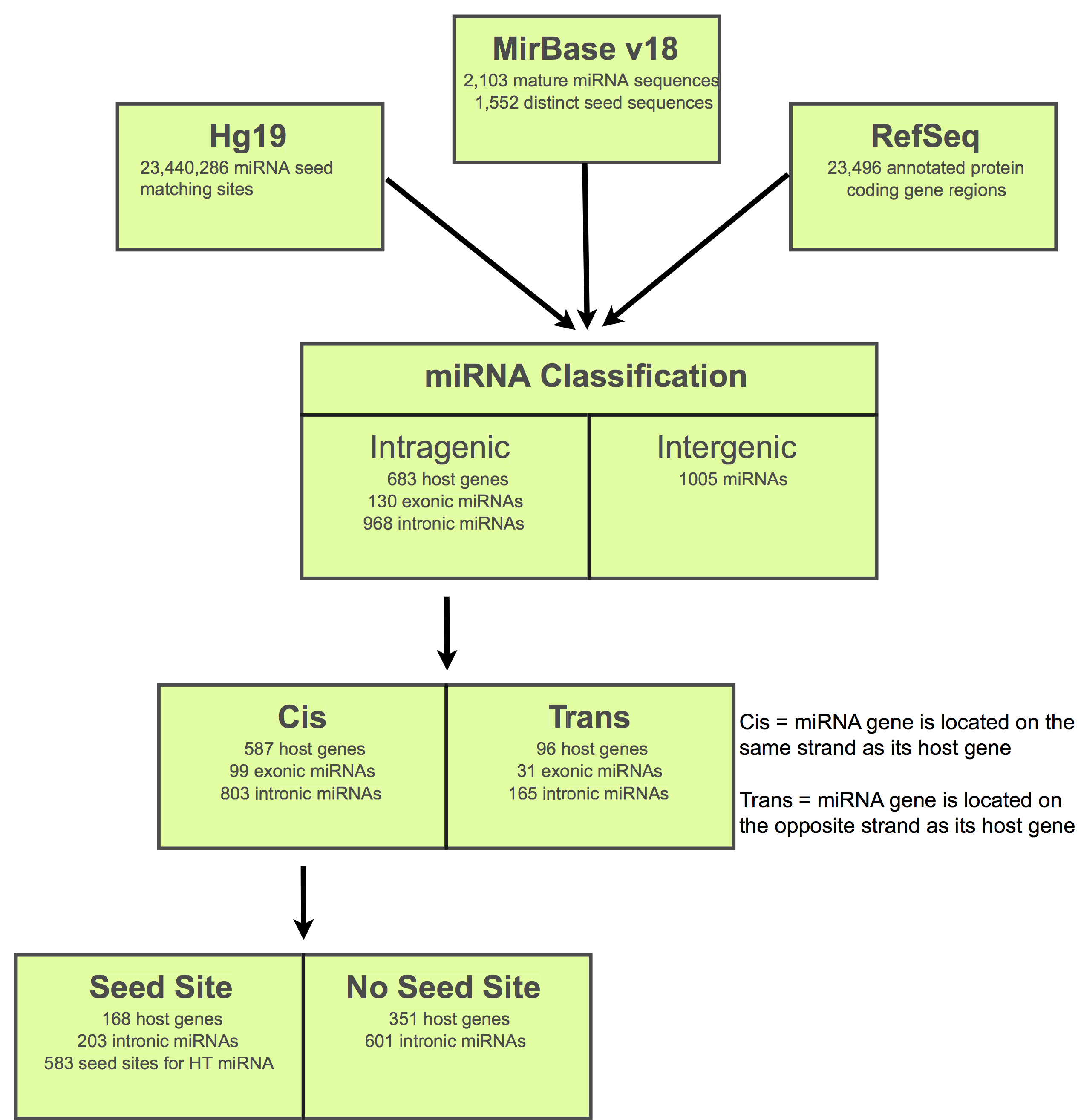

Supplement: S1 Fig — (TIFF) [file pone.0121507.s001.tiff]

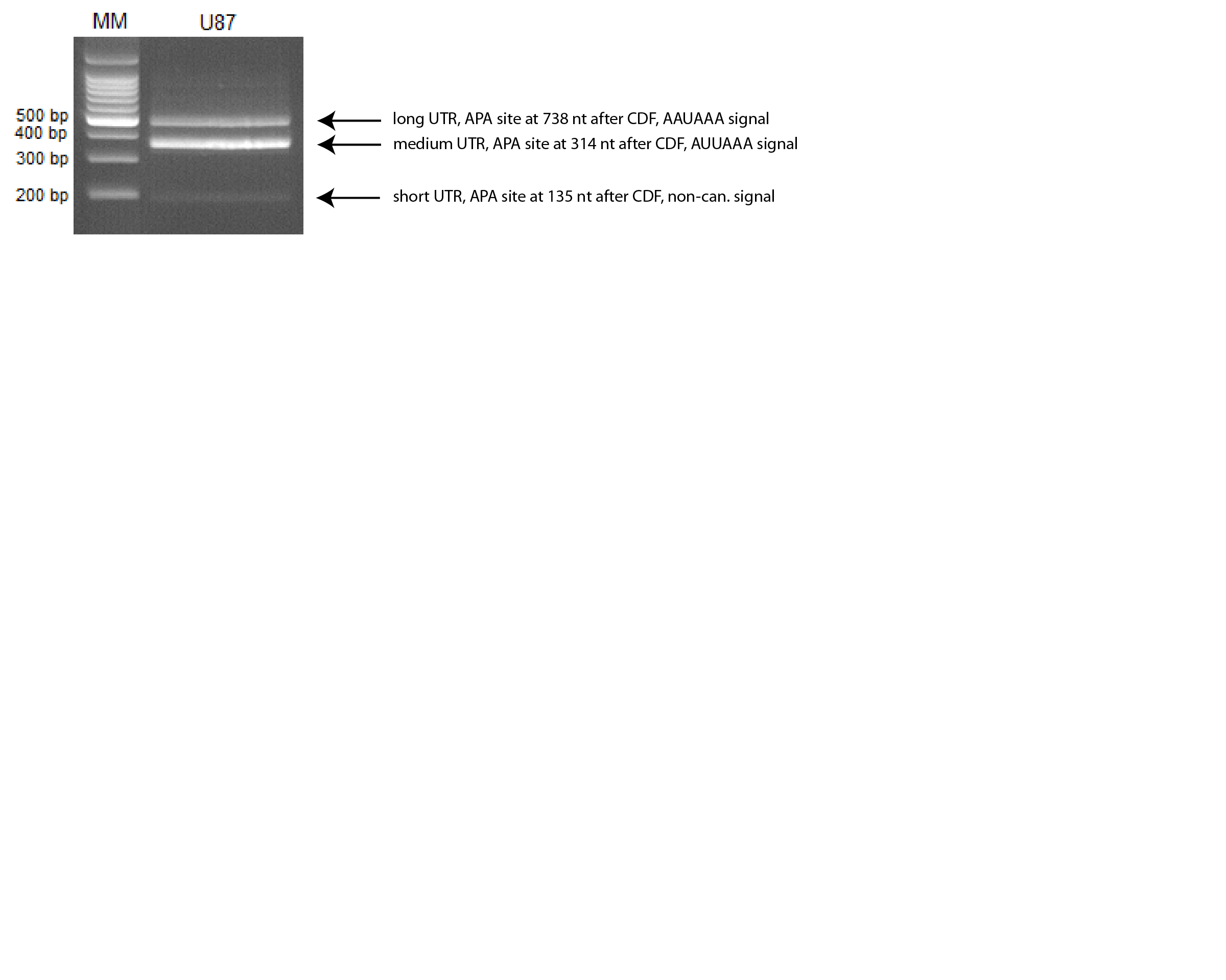

Supplement: S2 Fig — (TIFF) [file pone.0121507.s002.tiff]
