# Supplementary material for: Alternative Polyadenylation Allows Differential Negative Feedback of Human miRNA miR-579 on Its Host Gene ZFR
Source: PLoS One. 2015 Mar 23;10(3):e0121507. doi: 10.1371/journal.pone.0121507 (PMC4370670; doi:10.1371/journal.pone.0121507)
Supplement: S1 File — (PDF) [file pone.0121507.s003.pdf]

Supplementary Document S1. Extended Methods.

Vector construction

The psiCheck-2 Dual-Luciferase Vector (Promega) was used for the generation of ZFR and CPSF2 reporter constructs. Briefly, the ZFR 3'UTR or the CPSF2 3'UTR along with the predicted target site for miRNA-579 were PCR-amplified from human genomic DNA. Cycling conditions comprised an initial denaturation step of 95°C for 3 min, 35 cycles with 95°C for 30 s, 60°C for 45 s and 72°C for 30 s, and a final extension step of 72°C for 3 min. Oligonucleotides (synthesized by Metabion) are listed below. The PCR products were analysed by agarose gel electrophoresis, ligated into the pSC-B amp/kan vector (UltraBlunt PCR Cloning Kit, Stratagene) and finally subcloned into the *XhoI* and *PmeI* restriction sites of psiCheck-2 vector (Promega).

Site-directed mutagenesis of the miRNA-579 target sites was performed using the QuikChange Lightning Mutagenesis Kit (Stratagene) and the primers given below.

The wildtype and mutated reporter constructs were sequence-verified by MWG Biotech.

**Cloning**	
ZFR 3’UTR for	5’ CTCGAGATCCATTACCGCAAATGAGC 3’
ZFR 3’UTR rev	5’ GTTTAAACTGAAGCATACATCATTTCAAAGC 3’
CPSF2 3’UTR for	5’ CTCGAGGGACGCATTGGATTAGAAGG 3’
CPSF2 3’UTR rev	5‘ GTTTAAACTGCTTGGGTGACCTTAGGAG 3’
**Mutagenesis**	**(base exchanges are underlined)**
ZFR mut for	5’ AGCACTACAGTGAAATACACAAAAACCGAAATTCATATAATGAC 3’
ZFR mut rev	5’ GTCATTATATGAATTTCGGTTTTTGTGTATTTCACTGTAGTGCTG 3’
CPSF2 mut for	5’ GAGAACATTTTGCAAATGCTCAGGTGAGCATTCTATCTTTTGGC 3’
CPSF2 mut rev	5’ GCCAAAAGATAGAATGCTCACCTGAGCATTTGCAAAATGTTCTC 3’
**Real-time PCR**	
CPSF2 for	5‘ TGCCCTTTGCTATCTTCTCC 3‘
CPSF2 rev	5’ GCACTGCATCAATCTGGTGA 3’
	UPL probe #5
SDHA for	5’ GAGGCAGGGTTTAATACAGCA 3’
SDHA rev	5’ CCAGTTGTCCTCCTCCATGT 3’
	UPL probe #132
TBP for	5’ GAACATCATGGATCAGAACAACA 3’
TBP rev	5’ ATAGGGATTCCGGGAGTCAT 3’
	UPL probe #87
ZFR for	5’ TCAGAGGCAACAAGAAGCAC 3‘
ZFR rev	5‘ GCAGGAGCTGTGGACCTTAC 3‘
	UPL probe #77
ZFR long 3‘UTR for	5‘ CTTTTGCTTTGAAATGATGTATGC 3‘
ZFR short 3‘UTR for	5‘ TCTTCTCCACACACCCCATC 3‘
ZFR 3‘UTR linker rev	5‘ CGCTCGTGTCTTAATTATGCTG 3‘
**3’-RLM-RACE**	
3’-RACE outer primer	5' GCGAGCACAGAATTAATACGACT 3'
3’-RACE inner primer	5' CGCGGATCCGAATTAATACGACTCACTATAGG 3'
ZFR specific primer	5’ GCCCTTAATTATGGTGACAGTTC 3’
Poly(A)-linker	5’ TTTTTTTTTTTTTGGATATCACTCAGCATAATTAAGACACGAGCG 3’
